# Structure–Activity Relationship Development Efforts towards Peripherally Selective Analogs of the Cannabinoid Receptor Partial Agonist BAY 59-3074

**DOI:** 10.3390/molecules27175672

**Published:** 2022-09-02

**Authors:** George Amato, Vineetha Vasukuttan, Danni Harris, Lucas Laudermilk, Jennifer Lucitti, Scott Runyon, Rangan Maitra

**Affiliations:** Center for Drug Discovery, RTI International, P.O. Box 12194, Research Triangle Park, NC 27709-2194, USA

**Keywords:** CB1, cannabinoid, peripheral, CB2, partial, agonist, ligand

## Abstract

Selective modulation of peripheral cannabinoid receptors (CBRs) has potential therapeutic applications in medical conditions, including obesity, diabetes, liver diseases, GI disorders and pain. While there have been considerable efforts to produce selective antagonists or full agonists of CBRs, there has been limited reports on the development of partial agonists. Partial agonists targeting peripheral CBRs may have desirable pharmacological profiles while not producing centrally mediated dissociative effects. Bayer reported that BAY 59-3074 is a CNS penetrant partial agonist of both CB1 and CB2 receptors with efficacy in rat models of neuropathic and inflammatory pain. In this report, we demonstrate our efforts to synthesize analogs that would favor peripheral selectivity, while maintaining partial agonism of CB1. Our efforts led to the identification of a novel compound, which is a partial agonist of the human CB1 (hCB1) receptor with vastly diminished brain exposure compared to BAY 59-3074.

## 1. Introduction

Selective modulation of the peripheral G protein coupled cannabinoid receptors CB1 and CB2 has potential therapeutic applications in medical conditions, including obesity, diabetes, liver diseases, gastrointestinal (GI) disorders and pain [1,2,3]. There has been considerable effort to produce selective antagonists or full agonists of CBRs, but there have been limited reports on partial agonists, particularly peripherally selective partial agonists. Partial agonists of peripheral CBRs may have pharmacological profiles that mimic those of the partial agonist (−)-trans-Δ^9^-Tetrahydrocannabinol (THC), the principal psychoactive component of marijuana, but without the dissociative effects associated with activation of brain CB1 receptors [4,5]. Recently, a CB2-selective partial peripheral agonist was shown to have a protective effect in a mouse model of nephrotoxicity and to have a safe in vivo profile [6].

**Figure 1:**
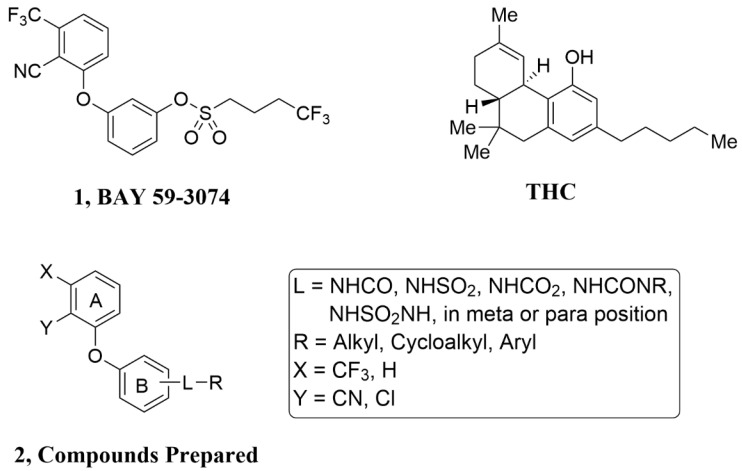
**THC**, BAY 59-3074 (**1**) and Compounds Prepared (**2**). To make (**1**) less CNS penetrating, the sulfonate linker (L) was swapped with a H-bond donating linker (amide, sulfonamide, carbamate, urea, and sulfamide. Changes to the trifluoromethyl (X), cyano (Y), and the trifluorobutyl group (R) of (**1**) were made to develop SAR with regard to potency and efficacy as described in Section 2.2.

Previously, Bayer Pharmaceuticals had described the novel trifluoro sulfonate chemotype BAY 38-7271, which is a full agonist of both CB1 and CB2 [7]. This brain penetrant compound was demonstrated to be neuroprotective in subsequent studies [8,9]. The compound BAY 59-3074 (**1**, Figure 1) is a CNS active partial agonist of both human and rat CB1 and CB2 receptors based on this chemotype [10]. It was shown that this compound has efficacy in rat models of neuropathic and inflammatory pain [10]. Activation of CB1 in the brain causes adverse psychiatric effects. Further, full agonists of CB1 rapidly induce tolerance. While tolerance to THC has been described, development of tolerance is typically protracted in users [11]. Additionally, full agonists of CB1 often produce other physiological ill-effects, as has been noted of late with synthetic cannabinoids including cardiac arrest and death [12,13]. Therefore, development of novel partial agonists will afford researchers the opportunity to better understand and contrast the effects of this class of compounds to those of full agonists. These partial agonists with limited brain penetration may also be used as leads for eventual medications development.

## 2. Results

### 2.1. Compound Design and Synthesis

Analyses of clinical compounds reveal that discovering a drug-like compound that is peripherally selective and orally bioavailable is favored with a topological polar surface area (TPSA) of 80–140 Å, a molecular weight (MW) of 450–600 Da, inclusion of one or two hydrogen bond donors (H donors) and the logarithm of its partition coefficient between n-octanol and water (cLogP) of less than five [14,15,16,17]. BAY 59-3074 has physical properties (H donors = 0, cLogP = 5.1, TPSA = 76, MW = 453) that are not far from the targeted values, with the most notable deficiency being a lack of hydrogen bond donors.

**Figure 2:**
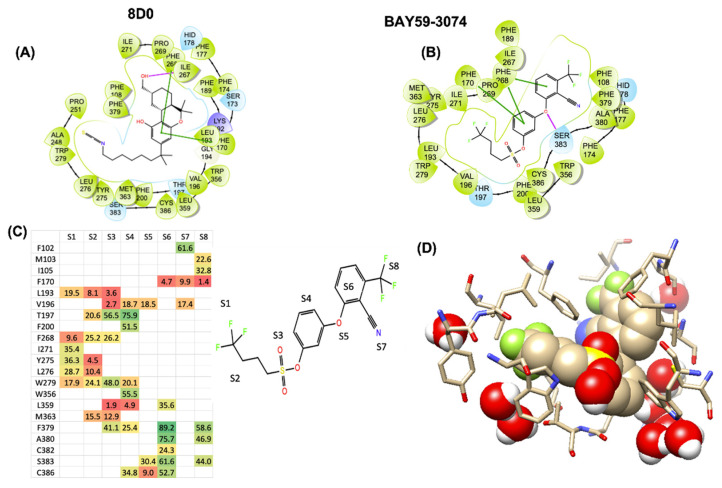
The LIGPLOT representations for the best Emodel scored induced-fit/GLIDE-XP docking poses of (**A**) agonist 8D0(AM841), and (**B**) compound **1** shows that they thread the largely hydrophobic CB1 binding site in a similar manner. The top MMGBSA-scored docking poses from Schrodinger’s induced fit/GLIDE-XP and Autodock VINA show π-stacking with F268^ECL2^ and F170^2.5^^7^ (**C**) The color-coded heat-map contact-analysis for the 850 ns molecular dynamic simulation reveals that while the sulfonyl (S3-region) of **1** spends significant percentages of total simulation time (heatmap) proximate to T197^3.33^ and W279^5.43^, direct H-bond/polar interactions with these polar residues is not persistent. Instead, bridged polar-residue—water H-bond stabilization is observed between these residues and the ligand sulfonyl. The heat-map spectrum is coded from green (high percentages) to red (low percentages). Panel (**D**) shows a 840 ns snapshot of **1** CB1 in DOPC/KCL/TIPS3P water from the 850 ns simulation highlighting these water bridging interactions.

Recent crystal structures of hCB1 and hCB2, coupled with docking studies of THC at the orthosteric binding site, show potential polar and nonpolar binding regions of importance [18,19,20,21,22]. We envisioned that **1** may be binding to hCB1 in such a way that the biphenyl ether is π-stacking with Phe268, the sulfonate ester alkyl chain is in the lipophilic channel, and the sulfonate linker is near to a polar residue. This was substantiated by docking studies of 1 with the orthosteric binding site of the hCB1 crystal structure (Figure 2). As noted in the initial published antagonist and agonist crystal/cyroEM structures of CB1, the binding site of CB1 is largely hydrophobic with only one or two polar sidechains [18,19,20,21]. We therefore conducted docking, induced fit, and molecular dynamics studies of **1** with the orthosteric binding site of the hCB1 crystal structure to identify potential interactions of the sulfonate linker with the receptor and facilitate identification of compounds with better drug-like properties while maintaining good hCB1 and hCB2 potency. Figure 2 shows that the best scoring GLIDE XP poses of the CB1 agonist 8D0 (the crystallographic ligand assigned name in the 5XR8 crystal structure corresponding to AM841: 6~{31}~{R},9~{R},10~{a}~{R})-9-(hydroxymethyl)-3-(8-isothiocyanato-2-methyl-octan-2-yl)-6,6-dimethyl-6~{a},7,8,9,10,10~{a}-hexahydrobenzo[c]chromen-1-ol) and **1** traverse the ligand binding site in a similar manner with π-stacking of the aromatic groups with one or more phenylalanines: F268^ECL2^, F170^2.57^, or F174^2.61^. The docked poses were found to be proximate to conserved aromatic toggle residues F200^3.36^ and W356^6.48^ in addition to W279^5.43^. In efforts to examine the temporal stability of a candidate ligand/GPCR-receptor initial docking/induced fit poses, we conducted 500–850 ns MD trajectories and illustrate our observations for **1** in Figure 2, panels C and D. The heatmap in Figure 2C color codes and shows the numerical percentage time that any of the atoms in each of the regions in **1** (molecular regions labeled S1 through S8, Figure 2) spend in contact within 4 Å binding site residues. This gives us a dynamic/temporal perspective akin to time-dependent pharmacophoric interactions. With an 850-nanosecond timespan, we see that the sulfonyl region of **1** does spend time in contact with T197^3.33^ and also spends some time getting close to the indole-NH of W279^5.43^ but it does not find robust direct hydrogen bonding. Instead, water percolation into the CB1 binding site through small fluctuations of the ligand leads to high occupancy of water molecules proximate to the sulfonyl. In Figure 2D, we show a structural snapshot at around 840 nanoseconds, with a water molecule forming a bridged hydrogen bond network linking the indole-NH of W279^5.43^ with one of the sulfonyl oxygens. The stabilization of transient water occupancy by polar residue sidechains leads to polar compensation for the negative charge density on the sulfonyl. These studies allowed us to design compounds based on computational observations.

By targeting modification of the sulfonate linker, we hypothesized that it would be possible to identify compounds with peripheral selectivity while maintaining good hCB1 and hCB2 potency. In this report, we explored introducing at least one hydrogen bond donor by exchange of the sulfonate linker with a sulfonamide, amide, carbamate, urea or sulfamide (**2**, Figure 1). To further reduce cLogP, we investigated replacing the trifluoromethyl group of arene A with hydrogen. Potency and efficacy were explored with alkyl, cycloalkyl and aryl groups attached to the linker. We also investigated the effect of changing the substitution pattern of the phenyl core (B) from *meta* to *para*.

**Scheme 1:**
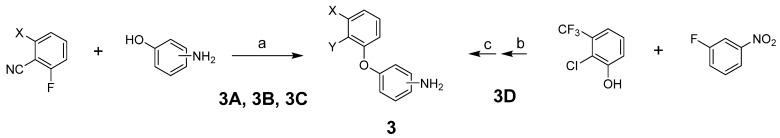
Reagents and conditions: (**a**) K_2_CO_3_, DMF, 80 °C; (**b**) K_2_CO_3_, DMA, 70 °C; (**c**) SnCl_2_·2H_2_O, EtOH, 50 °C.

To prepare the targeted compounds, we first synthesized the penultimate aminobiphenyl ethers **3** (Scheme 1). One of two methods was used, depending on whether Y was CN or Cl. These methods used a straightforward displacement of an aryl fluoride by a phenol (Reactions (a) and (b)). Where indicated, the fluoride displacement was followed by a nitro reduction using tin chloride (Reaction (c)). Intermediate **3D** was previously reported [23].

**Scheme 2:**
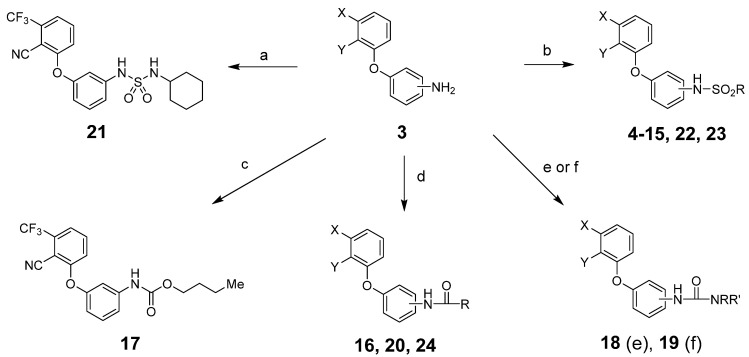
Reagents and conditions: (**a**) RNHSO_2_Cl, NEt_3_, THF, room temperature (rt); (**b**) RSO_2_Cl, NEt_3_, THF, rt; (**c**) RO_2_CCl, NEt_3_, THF, rt; (**d**) RCOCl, NEt_3_, THF, rt; (**e**) RNCO, NEt_3_, THF, rt; (**f**) (1) triphosgene, NaHCO_3_, CH_2_Cl_2_, water, rt; (2) RR’NH, NEt_3_, THF, rt. See Table 1 and Table 2 below for exact structures and in vitro data.

From intermediates **3**, we prepared sulfonamides, amides, carbamates, ureas and sulfamides using standard procedures as shown in Scheme 2 and detailed in the experimental section.

### 2.2. Pharmacological Characterization

All target compounds were evaluated in a functional cAMP assay for hCB1 (Table 1 and Table 2). To peripheralize 1, we investigated analogs in which the sulfonate linker was replaced with a sulfonamide. This adds a hydrogen bond donor, significantly lowers the cLogP and modestly increases TPSA, all changes that favor peripherally selective analogs. A patent filed by Bayer suggested that such compounds would be active in the CB1 agonist assay [23]. Table 1 shows the in vitro data for sulfonamide analogs of **1**. Compound **4** is the direct analog of **1** in which the sulfonate linker is replaced with a sulfonamide. This linker switch results in a favorable reduction in cLogP from 5.1 to 4.2 (see Supplemental Material, Table S1). We were encouraged to see that the hCB1 potency did not change much in the cAMP hCB1 assay. The efficacy, however, increased to provide full agonism. This unwanted result led us to investigate structure–activity relationships (SAR) to reduce hCB1 efficacy while retaining potency. Looking at the left side arene (A) of **4**, replacement of the CN with Cl or removal of the CF_3_ was found to have little effect on the hCB1 potency or efficacy (see **5** and **6**, Table 1, left section). The lack of improvement in efficacy led us to look at changes in other parts of the molecule. On the lipophilic sulfonamide group R, replacement of the CF_3_ of **4** with a methyl results in similar potency and modestly lower efficacy (see **7**, Table 1, left section). Replacement of the linear alkyl chain of **4** with a benzyl group provided the benzylsulfonamide **8**, which is similar in potency and efficacy to **4**. With the benzylsulfonamide, we once again checked replacement of the aryl CN with Cl (**9**) or the CF_3_ with hydrogen (**10**). Once again, these changes resulted in compounds with similar potency, but the efficacy was modestly reduced compared to **8**. Compound **10** was of interest because replacement of the trifluoromethyl group of arene A with hydrogen resulted in a significant drop in cLogP to 3.6, which could help to improve drug properties and attain peripheral selectivity, but the efficacy was still too high. The effect of chloro substitution of the benzylsulfonamide was examined. Surprisingly, *ortho* or *meta* substitution (**11** and **12**, Table 1, right section) resulted in loss of activity. Para-substitution (**13**), however, resulted in a compound that is about 10× more potent with modestly reduced efficacy, compared to the unsubstituted benzylsulfonamide **8**. Arenesulfonamides with either a *meta* or *para* trifluoromethoxy group (**14** and **15**, Table 1, right section) were found to be inactive. In summary, these results indicate that it would be difficult to find a sulfonamide with the desired profile of being a peripheral partial agonist of hCB1 receptors. Compounds **6** and **10** have good physical properties to favor peripheral selectivity, but like the other sulfonamides tested, they are full agonists of hCB1. 

In addition to sulfonamide analogs of **1**, we also investigated changing the linker to an amide, carbamate, urea or sulfamide. Each of these linkers adds at least one hydrogen bond donor, enhancing the possibility of peripheral selectivity. Direct replacement of the sulfonamide linker of the n-pentylsulfonamide **7** with an amide, carbamate or urea resulted in a loss of agonist activity in the cAMP hCB1 assay (**16–19**, Table 2, left section). However, conversion of the benzylsulfonamide **8** to the benzylamide **20** resulted in reasonable potency (~500 nM) but very low efficacy (<20%) in the cAMP hCB1 assay. Of significance, the cyclohexylsulfamide **21** is a partial agonist in the cAMP hCB1 assay (EC_50_ ~140 nM, 27%). Binding affinity (Ki) of this compound is ~1.5 µM as calculated using radioligand displacement of [^3^H]CP55940. Compound **21** has improved physical properties (TPSA = 91, cLogP = 4.2, H donors = 2) that favor peripheral selectivity and may thus represent a starting point for additional SAR investigations. These initial results show that the effects of changing the linker are dependent on what group is connected to it and may lead to compounds with the desired profile.

**Table 2:** Investigation of linkers and para-substitutions.

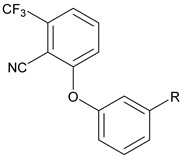			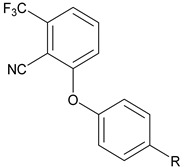		
**#**	**R**	**hCB1 cAMP** **EC_50_ (nM)** **Efficacy ^a^**	**#**	**R**	**hCB1 cAMP** **EC_50_ (nM)** **Efficacy ^a^**
**16**	NHCO(CH_2_)_4_Me	Inactive	**22**	NHSO_2_(CH_2_)_4_Me	Inactive
**17**	NHCO_2_(CH_2_)_3_Me	Inactive	**23**	NHSO_2_CH_2_Ph	Inactive
**18**	NHCONH(CH_2_)_3_Me	Inactive	**24**	NHCOCH_2_Ph	366 ± 136 36 ± 14%
**19**	NHCONMe(CH_2_)_3_Me	Inactive			
**20**	NHCOCH_2_Ph	474 ± 174 17 ± 3%			
**21**	NHSO_2_NH(c-Hex)	139 ± 24 27 ± 18%			

^a^ Efficacy was measured as a % of the maximum CP55940 (full agonist) signal in CHO cell membrane preparations overexpressing hCB1 receptor. Data reported as mean + SEM.

The effect of switching the substitution pattern of the phenyl core from *meta* to *para* was also investigated. The benzylsulfonamide **23** was inactive in the cAMP hCB1 assay. The benzylamide **24**, however, was a good partial agonist in the cAMP hCB1 assay (~370 nM, 36%). With a hydrogen bond donor to help aid peripheral selectivity, **24** represents another potential starting point for additional SAR studies and supports additional evaluation of compounds that are para-substituted.

### 2.3. Pharmacokinetic Evaluation of Brain Exposure in Mice

**Figure 3:**
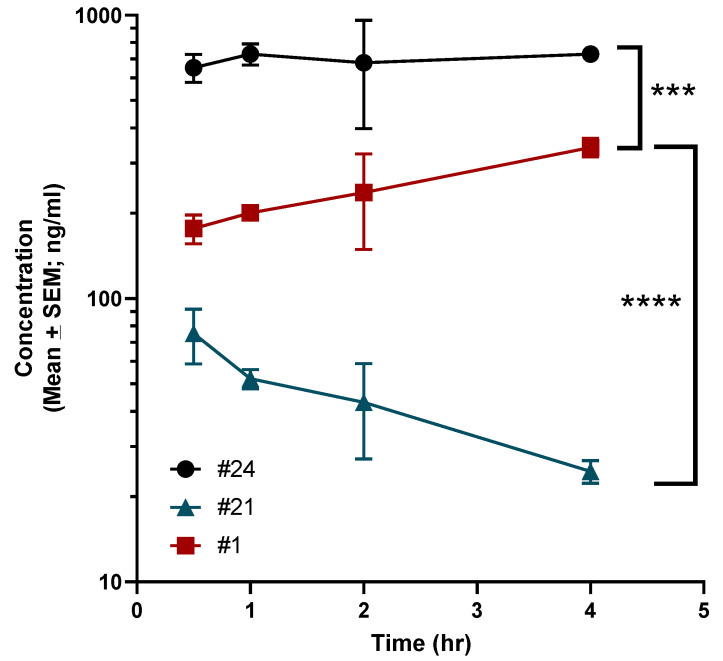
Pharmacokinetic evaluation of compounds **1**, **21** and **24** in mice. Mice were administered 3 mg/kg of each compound by IP injection as has been described in the Methods. The concentration of each compound in the brain was calculated using LC-MS at various time points. Data are presented as mean ± standard error. Statistical significance was determined using ANOVA with Dunnett’s multiple comparisons test. Significance indicated as **** *p* < 0.00001; *** *p* < 0.0001.

A brief pharmacokinetic (PK) study was undertaken in C57BL6 mice to assess brain levels of **1**, **21** and **24**. Animals were dosed with 3 mg/kg of each compound by intraperitoneal injection (IP). As demonstrated in Figure 3, compared to **1**, **21** had vastly reduced brain penetration with a maximum (Cmax) concentration of ~75 ng/mL with a brain:plasma Cmax ratio of 0.17. In comparison, **1** demonstrated gradual brain accumulation over the time points that were evaluated with a Cmax of ~341 ng/mL with a brain:plasma Cmax ratio of 0.40. However, **24** had significant brain accumulation with a Cmax of ~730 ng/mL with brain:plasma Cmax ratio of 0.79.

## 3. Discussion

There have been many reports that describe the synthesis and characterization of full agonists of cannabinoid receptors. These compounds may be useful for treating important diseases. However, there are certain disadvantages as well. First and foremost, activation of central CB1 receptors can lead to dissociative effects and both synthetic and natural cannabinoids are addictive. Rapid development of tolerance is also an issue with these full agonist compounds [24]. While tolerance to marijuana and THC is noted in heavy users, the amount of time required for tolerance to develop is longer than what is noted with full agonists. There is a paucity of synthetic CB1 ligands that are partial agonists. This is likely because THC is one and is remarkably effective in treating various conditions, at least preclinically with some positive results noted in clinical trials [25]. Indeed, synthetic analogs of THC are approved drugs and marijuana is approved for medical use in a number of states here in the United States. Of the limited number of partial agonists that have been characterized, **1** is a centrally acting partial agonist that was developed by Bayer and shown to effectively treat neuropathic and inflammatory pain in animal models [10]. Of these two indications, neuropathic pain is particularly difficult to manage. Most agents used for this purpose produce deleterious effects. Many preclinical and clinical studies indicate that activation of the CB1 receptor is an attractive strategy for neuropathic pain [26]. Therefore, partial agonists targeting CB1 that have little to no brain penetration are attractive because such ligands would limit liabilities associated with activation of brain CB1 receptors.

Structure–activity relationships of **1** would be useful in developing better compounds with limited CNS penetration. Recent crystal structures of hCB1 and docking studies with **1** indicate that the sulfonate linker is near to a polar region of the binding pocket and could be replaced with a polar hydrogen bond donating linker to facilitate development of peripherally selective analogs. Initial results showed this to be the case but controlling the level of efficacy is challenging. Effects on potency and efficacy are interdependent on the linker, the attached group, and the substitution pattern of the core. While a clear picture of the SAR to develop peripheral partial agonists of **1** did not emerge, we identified two hCB1 partial agonists with improved physical properties. Combining a change in the core substitution pattern from *meta* to *para* with replacement of the sulfonate linker with an amide resulted in the benzylamide **24** which has a hydrogen bond donor and is a partial agonist in the cAMP hCB1 assay. Replacement of the linker with a sulfamide and exchange of the alkyl chain with a cyclohexyl group resulted in the sulfamide **21**, also a partial agonist in the cAMP hCB1 assay. Compound **21** already has physical properties that strongly favor peripheral selectivity (TPSA = 91, cLogP = 4.2, H donors = 2). This was confirmed in PK studies wherein brain concentration of this compound was greatly diminished versus that of **1**. Additional SAR studies based on **21** and **24** may provide a map to compounds with the desired profile. The activity and efficacy of these compounds at hCB2 were not examined and will form the basis of future studies along with possible in vivo examination of effects.

## 4. Material and Methods

**Chemistry General.** Purity and characterization of compounds were established by a combination of MS, LC/MS, NMR, HPLC and TLC analytical techniques, as described below. ^1^H spectra were recorded on a Bruker Avance DPX-300 (300 MHz) spectrometer in chloroform-*d* (7.26 ppm) or methanol-*d_4_* (3.31 ppm) with tetramethylsilane (0.00 ppm) or solvent peaks as the internal reference unless otherwise noted. ^13^C spectra were recorded on a JEOP 400YH (100 MHz). Chemical shifts are reported in ppm relative to the solvent signal and coupling constant (*J*) values are reported in hertz (Hz). TLC was performed on EMD precoated silica gel 60 F254 plates. TLC spots were visualized with UV light or I_2_ detection. Low-resolution mass spectra were obtained using a single quadrupole PE Sciex API 150EX (ESI). Unless stated otherwise, all test compounds were at least 95% pure as determined by HPLC. HPLC method: a Waters 2695 Separation Module equipped with a Waters 2996 Photodiode Array Detector and a Phenomenex Synergi 4 μm Hydro-RP 80A C18 250 × 4.6 mm column using a flow rate of 1 mL/min starting with 1 min at 5% solvent B, followed by a 15 min gradient of 5–95% solvent B, followed by 9 min at 95% solvent B (solvent A, water with 0.1% TFA; solvent B, acetonitrile with 0.1% TFA and 5% water; absorbance monitored at 220 and 280 nm). LC/MS Instrument: an Agilent InfinityLab MSD single quadrupole mass spectrometer equipped with an API-ES and an Agilent Infinity II 1260 HPLC equipped with an Agilent Infinity 1260 variable wavelength detector and an Agilent Poroshell 120 SB-C18 2.7 μm 50 × 4.8 mm column. HPLC Method: With a flow rate of 1 mL/min, a 4.0 min gradient of 5–95% solvent B, followed by 2.5 min at 95% solvent B (solvent A, water with 0.1% formic acid; solvent B, acetonitrile with 0.1% formic acid and 5% water; absorbance monitored at 220 and 280 nm). MS Method: using atmospheric pressure ionization–electrospray, positive and negative ions were monitored in the range of 70–700 or 300–2000 (for compounds with a MW > 700).

**General Procedure A: Sulfonamides, Sulfamides, Carbamates, Amides and Ureas of 3 from Sulfonyl Chlorides, Sulfamoyl Chlorides, Carbamoyl Chlorides, Acid Chlorides and Isocyanates, Respectively**. To a solution of 3 (0.2 mmol, 1 equiv.) in THF (1 mL) was added the sulfonyl chloride, sulfamoyl chloride, carbamoyl chloride, acid chloride or isocyanate (0.24 mmol, 1.2 equiv.), followed by triethylamine (0.26 mmol, 1.3 equiv.). The mixture was stirred at rt for 15 h. Water (0.4 mL), ethyl acetate (3 mL) and then saturated NaHCO_3_ solution (0.8 mL) were added. After 10 min, the aqueous layer was removed. Celite (600 mg) was added to the organic layer, and the solvent was evaporated. Flash chromatography using silica gel with an ethyl acetate/hexanes gradient provided the purified sulfonamide, sulfamide, carbamate, amide, or urea.

**General Procedure B: Phenyl Ethers 3 from 2-Fluorobenzonitriles.** A mixture of 2-fluoro-6-trifluoromethylbenzonitrile or 2-fluorobenzonitrile (2 mmol), 3-aminophenol or 4-aminophenol (2.2 mmol, 1.1 equiv.), K_2_CO_3_ (6 mmol, 3 equiv.) and DMF (4 mL) was stirred at rt for 15 min and then at 80 °C for 4 h. Ethyl acetate (20 mL) was added, followed by water (4 mL) and brine (10 mL). After 10 min, the aqueous layer was removed, and the organic layer was washed with 0.8 M NaHCO_3_ solution (2 × 6 mL). Celite (3 g) was added to the organic layer and the solvent evaporated. Flash chromatography using silica gel with an ethyl acetate/hexanes gradient provided the purified phenyl ether **3**.

**2-(3-Aminophenoxy)-6-trifluoromethylbenzonitrile(3A).** The title compound was prepared by the general procedure B from 2-fluoro-6-trifluoromethylbenzonitrile (380 mg, 2.0 mmol), 3-aminophenol (240 mg, 1.1 equiv.), and K_2_CO_3_ (820 mg, 3 equiv.) to provide 550 mg (99%) of a colorless oil. R_f_ = 0.35 (40% ethyl acetate/hexanes; UV active). LC/MS (*m/z*) 279.0 (M+1), >95% at 4.36 min.

**2-(4-Aminophenoxy)-6-trifluoromethylbenzonitrile(3B).** The title compound was prepared by the general procedure B from 2-fluoro-6-trifluoromethylbenzonitrile (380 mg, 2.0 mmol), 4-aminophenol (240 mg, 1.1 equiv.), and K_2_CO_3_ (820 mg, 3 equiv.) to provide 451 mg (82%) of a tan crystalline solid. R_f_ = 0.24 (40% ethyl acetate/hexanes; UV active). LC/MS (*m/z*) 279.0 (M+1), 85% at 3.94 min.

**2-(3-Aminophenoxy)benzonitrile(3C).** The title compound was prepared by the general procedure B from 2-fluorobenzonitrile (0.17 mL, 1.5 mmol), 3-aminophenol (180 mg, 1.1 equiv.), and K_2_CO_3_ (620 mg, 3 equiv.) to provide 270 mg (86%) of a colorless oil. R_f_ = 0.40 (40% ethyl acetate/hexanes; UV active). LC/MS (*m/z*) 211.0 (M+1), >97% at 3.84 min.

**3-[2-Chloro-3-(trifluoromethyl)phenoxy]benzenamine(3D).** A mixture of 2-chloro-3-trifluoromethylphenol (0.27 mL, 2.0 mmol), 3-fluoronitrobenzene (0.26 mL, 1.2 equiv.), K_2_CO_3_ (820 mg, 3 equiv.) and DMA (4 mL) was stirred at rt for 15 min and then at 70 °C for 2 h. Ethyl acetate (20 mL) was added, followed by water (4 mL) and brine (10 mL). After 10 min, the aqueous layer was removed, and the organic layer was washed with brine (2 × 6 mL). Celite (3 g) was added to the organic layer and the solvent evaporated. Flash chromatography using silica gel with an ethyl acetate/hexanes gradient provided 620 mg of a yellow oil. R_f_ = 0.44 (20% ethyl acetate/hexanes; UV active). To the solution of the obtained oil in ethanol (6 mL) was added SnCl_2_ dihydrate (2.2 g, 5 equiv.), and the mixture was heated at 50 °C for 15 h. Most of the solvent was evaporated (rotary evaporator) and CH_2_Cl_2_ (20 mL) was added, followed by slow addition of saturated NaHCO_3_ solution (20 mL, vigorous gas evolution). Then, 6 N NaOH (1 mL) was added, and the mixture was stirred vigorously for 30 min. Water (20 mL) was added and the layers separated (a solid is removed with the aqueous layer). The aqueous layer was extracted with CH_2_Cl_2_ (2 × 10 mL). Celite (3 g) was added to the combined organic layers and the solvent evaporated. Flash chromatography using silica gel with an ethyl acetate/hexanes gradient provided 459 mg (80%) of a clear oil. R_f_ = 0.26 (20% ethyl acetate/hexanes; UV active). LC/MS (*m/z*) 288.0 (M+1), >95% at 4.69 min.

**N-{3-[2-Cyano-3-(trifluoromethyl)phenoxy]phenyl}-4,4,4-trifluorobutane-1-sulfonamide(4).** The title compound was prepared by the general procedure A from **3A** (50 mg, 0.18 mmol), 4,4,4-trifluorobutane-1-sulfonyl chloride (46 mg, 1.2 equiv.), and pyridine (0.030 mL, 2 equiv.) to provide 42 mg (53%) of an off-white crystalline solid, mp 96–97 °C. R_f_ = 0.34 (40% ethyl acetate/hexanes; UV active). ^1^H NMR (300 MHz, CDCl_3_) δ 7.57–7.73 (m, 1H), 7.51 (d, *J* = 8.1 Hz, 1H), 7.41 (t, *J* = 8.4 Hz, 1H), 7.13 (d, *J* = 8.3 Hz, 1H), 7.04–7.08 (m, 2H), 6.89 (d, *J* = 8.5 Hz, 1H), 6.83 (s, 1H), 3.24 (t, *J* = 7.4 Hz, 2H), 2.21–2.43 (m, 2H), 2.05–2.20 (m, 2H). MS (*m/z*) 451.3 (M-1). HPLC 98% at 15.80 min.

**N-{3-[2-Chloro-3-(trifluoromethyl)phenoxy]phenyl}-4,4,4-trifluorobutane-1-sulfonamide(5).** The title compound was prepared by the general procedure A from **3D** (44 mg, 0.15 mmol), 4,4,4-trifluorobutane-1-sulfonyl chloride (38 mg, 1.2 equiv.), and triethylamine (0.027 mL, 1.3 equiv.) to provide 43 mg (62%) of a colorless oil. R_f_ = 0.36 (30% ethyl acetate/hexanes; UV active). ^1^H NMR (300 MHz, CDCl_3_) δ 7.46–7.63 (m, 1H), 7.28–7.42 (m, 2H), 7.14–7.24 (m, 1H), 6.95 (d, *J* = 7.7 Hz, 1H), 6.86 (s, 1H), 6.75 (d, *J* = 7.7 Hz, 1H), 6.58 (s, 1H), 3.20 (br s, 2H), 2.18–2.41 (m, 2H), 2.06–2.17 (m, 2H). MS (*m/z*) 460.3 (M-1). HPLC 96% at 17.00 min.

**N-[3-(2-Cyanophenoxy)phenyl]-4,4,4-trifluorobutane-1-sulfonamide(6).** The title compound was prepared by the general procedure A from **3C** (32 mg, 0.15 mmol), 4,4,4-trifluorobutane-1-sulfonyl chloride (38 mg, 1.2 equiv.), and triethylamine (0.027 mL, 1.3 equiv.) to provide 17 mg (30%) of a colorless oil. R_f_ = 0.25 (30% ethyl acetate/hexanes; UV active). ^1^H NMR (300 MHz, CDCl_3_) δ 7.68 (d, *J* = 7.7 Hz, 1H), 7.48–7.59 (m, 1H), 7.31–7.43 (m, 1H), 7.16–7.25 (m, 1H), 6.92–7.07 (m, 3H), 6.86 (d, *J* = 9.0 Hz, 1H), 6.62 (s, 1H), 3.22 (t, *J* = 7.4 Hz, 2H), 2.19–2.41 (m, 2H), 2.06–2.19 (m, 2H). MS (*m/z*) 383.3 (M-1). HPLC > 99% at 14.95 min.

**N-{3-[2-Cyano-3-(trifluoromethyl)phenoxy]phenyl}pentane-1-sulfonamide(7).** The title compound was prepared by the general procedure A from **3A** (42 mg, 0.15 mmol), *n*-pentane-1-sulfonyl chloride (0.026 mL, 1.2 equiv.), and pyridine (0.027 mL, 2.2 equiv.) to provide 60 mg (97%) of a colorless oil. R_f_ = 0.49 (40% ethyl acetate/hexanes; UV active). ^1^H NMR (300 MHz, CDCl_3_) δ 7.55–7.70 (m, 1H), 7.50 (d, *J* = 8.1 Hz, 1H), 7.39 (t, *J* = 8.5 Hz, 1H), 7.12 (d, *J* = 8.1 Hz, 1H), 6.98–7.08 (m, 2H), 6.87 (d, *J* = 8.5 Hz, 1H), 6.75 (s, 1H), 3.15 (t, *J* = 7.5 Hz, 2H), 1.74–1.91 (m, 2H), 1.21–1.47 (m, 4H), 0.89 (t, *J* = 7.4 Hz, 3H). MS (*m/z*) 413.4 (M+1), 411.4 (M-1). HPLC > 99% at 16.46 min.

**N-{3-[2-Cyano-3-(trifluoromethyl)phenoxy]phenyl}-1-phenylmethanesulfonamide(8).** The title compound was prepared by the general procedure A from **3A** (45 mg, 0.15 mmol), 1-phenylmethanesulfonyl chloride (35 mg, 1.2 equiv.), and triethylamine (0.027 mL, 1.3 equiv.) to provide 23 mg (36%) of an off-white crystalline solid, mp 131–132 °C. R_f_ = 0.48 (40% ethyl acetate/hexanes; UV active). ^1^H NMR (300 MHz, CDCl_3_) δ 7.62 (br s, 1H), 7.52 (br s, 1H), 7.36 (br s, 6H), 7.09 (d, *J* = 7.4 Hz, 1H), 6.89–6.98 (m, 1H), 6.84 (br s, 2H), 6.48 (s, 1H), 4.39 (s, 2H). MS (*m/z*) 431.2 (M-1). HPLC > 99% at 15.87 min.

**N-{3-[2-Chloro-3-(trifluoromethyl)phenoxy]phenyl}-1-phenylmethanesulfonamide(9).** The title compound was prepared by the general procedure A from **3D** (44 mg, 0.15 mmol), 1-phenylmethanesulfonyl chloride (35 mg, 1.2 equiv.), and triethylamine (0.027 mL, 1.3 equiv.) to provide 23 mg (35%) of an off-white crystalline solid, mp 129–130 °C. R_f_ = 0.36 (30% ethyl acetate/hexanes; UV active). ^1^H NMR (300 MHz, CDCl_3_) δ 7.48–7.60 (m, 1H), 7.21–7.46 (m, 8H), 6.89 (d, *J* = 7.5 Hz, 1H), 6.69–6.77 (m, 2H), 6.32 (s, 1H), 4.36 (s, 2H). MS (*m/z*) 440.4 (M-1). HPLC > 99% at 17.25 min.

**N-[3-(2-Cyanophenoxy)phenyl]-1-phenylmethanesulfonamide(10).** The title compound was prepared by the general procedure A from **3C** (32 mg, 0.15 mmol), 1-phenylmethanesulfonyl chloride (35 mg, 1.2 equiv.), and triethylamine (0.027 mL, 1.3 equiv.) to provide 12 mg (22%) of a colorless oil. R_f_ = 0.25 (30% ethyl acetate/hexanes; UV active). ^1^H NMR (300 MHz, CDCl_3_) δ 7.69 (d, *J* = 7.7 Hz, 1H), 7.48–7.59 (m, 1H), 7.27–7.41 (m, 6H), 7.15–7.24 (m, 1H), 6.89–7.00 (m, 2H), 6.79–6.88 (m, 2H), 6.34 (s, 1H), 4.38 (s, 2H). MS (*m/z*) 363.4 (M-1). HPLC > 98% at 15.00 min.

**1-(2-Chlorophenyl)-N-{3-[2-cyano-3-(trifluoromethyl)phenoxy]phenyl}methanesulfonamide(11).** The title compound was prepared by the general procedure A from **3A** (42 mg, 0.15 mmol), 1-(2-chlorophenyl)methanesulfonyl chloride (41 mg, 1.2 equiv.), and pyridine (0.036 mL, 3 equiv.) to provide 43 mg (61%) of a white crystalline solid, mp 121–122 °C. R_f_ = 0.24 (30% ethyl acetate/hexanes; UV active). ^1^H NMR (300 MHz, CDCl_3_) δ 7.55–7.69 (m, 1H), 7.49 (d, *J* = 7.5 Hz, 1H), 7.38–7.46 (m, 1H), 7.24–7.37 (m, 3H), 7.06 (d, *J* = 8.5 Hz, 1H), 6.94 (d, *J* = 8.7 Hz, 1H), 6.91 (s, 1H), 6.82 (s, 1H), 6.80 (d, *J* = 8.7 Hz, 1H), 4.63 (s, 2H). MS (*m/z*) 465.2 (M-1). HPLC 96% at 16.24 min.

**1-(3-Chlorophenyl)-N-{3-[2-cyano-3-(trifluoromethyl)phenoxy]phenyl}methanesulfonamide(12).** The title compound was prepared by the general procedure A from **3A** (42 mg, 0.15 mmol), 1-(3-chlorophenyl)methanesulfonyl chloride (41 mg, 1.2 equiv.), and pyridine (0.036 mL, 3 equiv.) to provide 46 mg (66%) of an off-white crystalline solid, mp 144–145 °C. R_f_ = 0.28 (30% ethyl acetate/hexanes; UV active). ^1^H NMR (300 MHz, CDCl_3_) δ 7.63 (t, *J* = 8.1 Hz, 1H), 7.51 (d, *J* = 7.9 Hz, 1H), 7.28–7.45 (m, 3H), 7.07–7.21 (m, 2H), 6.82–7.02 (m, 3H), 6.69 (s, 1H), 4.35 (s, 2H). MS (*m/z*) 465.2 (M-1). HPLC 96% at 16.38 min.

**1-(4-Chlorophenyl)-N-{3-[2-cyano-3-(trifluoromethyl)phenoxy]phenyl}methanesulfonamide(13).** The title compound was prepared by the general procedure A from **3A** (42 mg, 0.15 mmol), 1-(4-chlorophenyl)methanesulfonyl chloride (41 mg, 1.2 equiv.), and pyridine (0.036 mL, 3 equiv.) to provide 45 mg (64%) of a white amorphous solid, mp 115–116 °C. R_f_ = 0.27 (30% ethyl acetate/hexanes; UV active). ^1^H NMR (300 MHz, CDCl_3_) δ 7.58–7.71 (m, 1H), 7.52 (d, *J* = 7.5 Hz, 1H), 7.35–7.43 (m, 1H), 7.31 (d, *J* = 8.2 Hz, 2H), 7.20 (d, *J* = 8.2 Hz, 2H), 7.12 (d, *J* = 8.5 Hz, 1H), 6.90–7.00 (m, 2H), 6.86 (d, *J* = 7.4 Hz, 1H), 6.69 (s, 1H), 4.26–4.44 (m, 2H). MS (*m/z*) 465.2 (M-1). HPLC 95% at 16.47 min.

**N-{3-[2-Cyano-3-(trifluoromethyl)phenoxy]phenyl}-3-(trifluoromethoxy)benzene-1-sulfonamide(14).** The title compound was prepared by the general procedure A from **3A** (42 mg, 0.15 mmol), 1-(3-trifluoromethoxyphenyl)methanesulfonyl chloride (0.032 mL, 1.1 equiv.), and triethylamine (0.025 mL, 1.2 equiv.) to provide 55 mg (73%) of a white crystalline solid, mp 120–121 °C. R_f_ = 0.26 (30% ethyl acetate/hexanes; UV active). ^1^H NMR (300 MHz, CDCl_3_) δ 7.73 (d, *J* = 7.7 Hz, 1H), 7.39–7.68 (m, 5H), 7.28–7.38 (m, 1H), 6.83–7.06 (m, 4H), 6.77 (s, 1H). MS (*m/z*) 501.6 (M-1). HPLC > 99% at 16.74 min.

**N-{3-[2-Cyano-3-(trifluoromethyl)phenoxy]phenyl}-4-(trifluoromethoxy)benzene-1-sulfonamide(15).** The title compound was prepared by the general procedure A from **3A** (42 mg, 0.15 mmol), 1-(4-trifluoromethoxyphenyl)methanesulfonyl chloride (0.032 mL, 1.1 equiv.), and triethylamine (0.025 mL, 1.2 equiv.) to provide 47 mg (62%) of a white crystalline solid, mp 164–165 °C. R_f_ = 0.28 (40% ethyl acetate/hexanes; UV active). ^1^H NMR (300 MHz, CDCl_3_) δ 7.85 (d, *J* = 8.5 Hz, 2H), 7.54–7.68 (m, 1H), 7.50 (d, *J* = 7.5 Hz, 1H), 7.28–7.41 (m, 3H), 7.00 (d, *J* = 7.7 Hz, 1H), 6.83–6.97 (m, 3H), 6.80 (s, 1H). MS (*m/z*) 501.4 (M-1). HPLC > 99% at 16.80 min.

**N-{3-[2-Cyano-3-(trifluoromethyl)phenoxy]phenyl}hexanamide(16).** The title compound was prepared by the general procedure A from **3A** (42 mg, 0.15 mmol), *n*-pentanecarbonyl chloride (0.025 mL, 1.2 equiv.), and triethylamine (0.027 mL, 1.3 equiv.) to provide 56 mg (99%) of a white crystalline solid, mp 95–96 °C. R_f_ = 0.46 (40% ethyl acetate/hexanes; UV active). ^1^H NMR (300 MHz, CDCl_3_) δ 7.50–7.62 (m, 2H), 7.45 (d, *J* = 7.7 Hz, 1H), 7.32–7.40 (m, 1H), 7.22 (br s, 2H), 7.12 (d, *J* = 8.5 Hz, 1H), 6.85 (d, *J* = 6.6 Hz, 1H), 2.36 (t, *J* = 7.5 Hz, 2H), 1.67–1.76 (m, 2H), 1.31–1.40 (m, 4H), 0.91 (t, *J* = 7.5 Hz, 3H). MS (*m/z*) 377.3 (M+1), 375.3 (M-1). HPLC > 99% at 16.86 min.

**Butyl N-{3-[2-Cyano-3-(trifluoromethyl)phenoxy]phenyl}carbamate(17).** The title compound was prepared by the general procedure A from **3A** (42 mg, 0.15 mmol), n-butyl chloroformate (0.021 mL, 1.2 equiv.), and triethylamine (0.027 mL, 1.3 equiv.) to provide 44 mg (78%) of a colorless oil. R_f_ = 0.24 (30% ethyl acetate/hexanes; UV active). ^1^H NMR (300 MHz, CDCl_3_) δ 7.57 (t, *J* = 7.7 Hz, 1H), 7.45 (d, *J* = 7.7 Hz, 1H), 7.30–7.41 (m, 2H), 7.05–7.19 (m, 2H), 6.80 (dd, *J* = 8.1, 1.5 Hz, 1H), 6.68 (s, 1H), 4.16 (t, *J* = 7.4 Hz, 2H), 1.59–1.73 (m, 2H), 1.33–1.47 (m, 2H), 0.95 (t, *J* = 7.4 Hz, 3H). MS (*m/z*) 379.2 (M+1), 377.3 (M-1). HPLC > 99% at 17.21 min.

**3-Butyl-1-{3-[2-cyano-3-(trifluoromethyl)phenoxy]phenyl}urea(18).** The title compound was prepared by the general procedure A from **3A** (42 mg, 0.15 mmol), n-butyl isocyanate (0.021 mL, 1.2 equiv.), and triethylamine (0.025 mL, 1.2 equiv.) to provide 51 mg (90%) of a colorless oil. R_f_ = 0.23 (40% ethyl acetate/hexanes; UV active). ^1^H NMR (300 MHz, CDCl_3_) δ 7.56 (t, *J* = 7.7 Hz, 1H), 7.43 (d, *J* = 7.7 Hz, 1H), 7.27–7.36 (m, 2H), 7.02–7.19 (m, 2H), 6.79 (s, 1H), 6.73 (d, *J* = 7.9 Hz, 1H), 4.94 (br s, 1H), 3.16–3.32 (m, 2H), 1.43–1.55 (m, 2H), 1.27–1.42 (m, 2H), 0.92 (t, *J* = 7.2 Hz, 3H). MS (*m/z*) 378.3 (M+1), 376.2 (M-1). HPLC > 96% at 15.68 min.

**3-Butyl-1-{3-[2-cyano-3-(trifluoromethyl)phenoxy]phenyl}-3-methylurea(19).** To an ice-cold solution of **3A** (42 mg, 0.15 mmol) in CH_2_Cl_2_ (1 mL) was added NaHCO_3_ (62 mg, 5 equiv.), followed by saturated NaHCO_3_ solution (0.3 mL). Triphosgene (45 mg, 1 equiv.) was added, and after 10 min, the ice bath was removed, and the mixture was stirred at rt for 1 h (gas evolution). Water (0.6 mL) was added and after 10 min, the aqueous layer was removed. The organic layer dried with sodium sulfate (20 min) and filtered. Toluene (0.5 mL) was added and most of the solvent evaporated. THF (1 mL) was added, and the mixture cooled in an ice bath. N-Methyl-n-butylamine (0.053 mL, 3 equiv.) was added, followed by triethylamine (0.042 mL, 2 equiv.). The mixture was stirred at rt for 20 h. Ethyl acetate (3 mL) was added, followed by saturated NaHCO_3_ solution (0.8 mL) and water (0.4 mL). After 10 min, the aqueous layer was removed. Celite (600 mg) was added to the organic layer and the solvent was evaporated. Flash chromatography using silica gel with an ethyl acetate/hexanes gradient provided 58 mg (99%) of a colorless oil. R_f_ = 0.22 (40% ethyl acetate/hexanes; UV active). ^1^H NMR (300 MHz, CDCl_3_) δ 7.55 (t, *J* = 7.7 Hz, 1H), 7.37–7.47 (m, 2H), 7.28–7.37 (m, 1H), 7.05–7.20 (m, 2H), 6.70–6.84 (m, 1H), 6.41 (s, 1H), 3.34 (t, *J* = 7.4 Hz, 2H), 3.01 (s, 3H), 1.48–1.69 (m, 2H), 1.28–1.45 (m, 2H), 0.95 (t, *J* = 7.4 Hz, 3H). MS (*m/z*) x (M+1). HPLC > 99% at 16.33 min.

**N-{3-[2-Cyano-3-(trifluoromethyl)phenoxy]phenyl}-2-phenylacetamide(20).** The title compound was prepared by the general procedure A from **3A** (42 mg, 0.15 mmol), phenylacetyl chloride (0.024 mL, 1.2 equiv.), and triethylamine (0.027 mL, 1.3 equiv.) to provide 61 mg (100%) of a white crystalline solid, mp 136–137 °C. R_f_ = 0.34 (40% ethyl acetate/hexanes; UV active). ^1^H NMR (300 MHz, CDCl_3_) δ 7.55 (br s, 1H), 7.30–7.49 (m, 8H), 7.12–7.22 (m, 2H), 7.00–7.11 (m, 1H), 6.84 (br s, 1H), 3.74 (s, 2H). MS (*m/z*) 397.4 (M+1), 395.5 (M-1). HPLC > 99% at 16.07 min.

**N-Cyclohexyl({3-[2-cyano-3-(trifluoromethyl)phenoxy]phenyl}m amino)sulfonamide(21).** The title compound was prepared by the general procedure A from **3A** (42 mg, 0.15 mmol), cyclohexylsulfamoyl chloride (45 mg, 1.5 equiv.), and triethylamine (0.027 mL, 1.3 equiv.) to provide 63 mg (96%) of a white amorphous solid, mp 199–200 °C. R_f_ = 0.46 (40% ethyl acetate/hexanes; UV active). ^1^H NMR (300 MHz, CDCl_3_) δ 7.53–7.70 (m, 1H), 7.47 (d, *J* = 7.5 Hz, 1H), 7.30–7.42 (m, 1H), 7.08 (d, *J* = 8.5 Hz, 1H), 7.00 (br s, 3H), 6.81 (d, *J* = 7.5 Hz, 1H), 4.66 (d, *J* = 6.8 Hz, 1H), 3.13–3.38 (m, 1H), 1.77–1.96 (m, 2H), 1.65 (br s, 2H), 1.47–1.59 (m, 1H), 1.06–1.37 (m, 5H). ^13^C NMR (100 MHz, CDCl_3_) δ 161.2, 155.3, 139.7, 134.7, 134.2, 131.2, 120.6, 120.2, 115.8, 115.3, 112.3, 110.6, 101.1, 53.5, 33.8, 25.2, 24.7. MS (*m/z*) 438.3 (M-1). HPLC > 99% at 16.17 min.

**N-{4-[2-Cyano-3-(trifluoromethyl)phenoxy]phenyl}pentane-1-sulfonamide(22).** The title compound was prepared by the general procedure A from **3B** (42 mg, 0.15 mmol), *n*-pentane-1-sulfonyl chloride (0.026 mL, 1.2 equiv.), and triethylamine (0.027 mL, 1.3 equiv.) to provide 53 mg (86%) of a white crystalline solid, mp 94–95 °C. R_f_ = 0.49 (40% ethyl acetate/hexanes; UV active). ^1^H NMR (300 MHz, CDCl_3_) δ 7.59 (t, *J* = 8.1 Hz, 1H), 7.48 (d, *J* = 7.7 Hz, 1H), 7.29 (d, *J* = 8.9 Hz, 2H), 7.11 (d, *J* = 9.0 Hz, 2H), 7.07 (d, *J* = 8.7 Hz, 1H), 6.49 (s, 1 H), 2.96–3.21 (m, 2H), 1.77–1.94 (m, 2H), 1.24–1.48 (m, 4H), 0.90 (t, *J* = 7.4 Hz, 3H). MS (*m/z*) 413.1 (M+1), 411.3 (M-1). HPLC > 99% at 16.38 min.

**N-{4-[2-Cyano-3-(trifluoromethyl)phenoxy]phenyl}-1-phenylmethanesulfonamide(23).** The title compound was prepared by the general procedure A from **3B** (42 mg, 0.15 mmol), 1-phenylmethanesulfonyl chloride (35 mg, 1.2 equiv.), and triethylamine (0.027 mL, 1.3 equiv.) to provide 28 mg (43%) of an off-white crystalline solid, mp 166–167 °C. R_f_ = 0.44 (40% ethyl acetate/hexanes; UV active). ^1^H NMR (300 MHz, CDCl_3_) δ 7.55–7.68 (m, 1H), 7.48 (d, *J* = 7.7 Hz, 1H), 7.27–7.42 (m, 6H), 7.15 (d, *J* = 7.9 Hz, 2H), 7.00–7.13 (m, 3H), 6.47 (s, 1H), 4.37 (s, 2H). MS (*m/z*) 431.2 (M-1). HPLC > 99% at 15.89 min.

**N-{4-[2-Cyano-3-(trifluoromethyl)phenoxy]phenyl}-2-phenylacetamide(24).** The title compound was prepared by the general procedure A from **3B** (42 mg, 0.15 mmol), phenylacetyl chloride (0.024 mL, 1.2 equiv.), and triethylamine (0.027 mL, 1.3 equiv.) to provide 49 mg (82%) of a white crystalline solid, mp 200–201 °C. R_f_ = 0.31 (40% ethyl acetate/hexanes; UV active). ^1^H NMR (300 MHz, CDCl_3_) δ 7.46–7.60 (m, 3H), 7.27–7.45 (m, 6H), 7.19 (br s, 1H), 6.93–7.11 (m, 3H), 3.77 (s, 2H). MS (*m/z*) 397.3 (M+1), 395.4 (M-1). HPLC > 99% at 15.91 min.

### 4.1. cAMP Accumulation Assay

The cAMP assays were performed in Chinese Hamster Ovary (CHO) cells (Perkin Elmer, Cat # ES-110-C) stably expressing the human CB1 receptor (hCB1) cultured under standard cell culture conditions (37 °C, 5% carbon dioxide, DMEM media with 1% Penicillin/Streptomycin and 400 µg/mL G418) using the Lance™ assay kit and manufacturer’s instructions were closely followed (Perkin Elmer). In brief, stimulation buffer containing 1X Hank’s Balanced Salt Solution (HBSS), 5 mM HEPES, 0.1% BSA stabilizer, and 0.5 mM final IBMX was prepared and titrated to pH 7.4 at rt. Serial dilutions of the test compounds and 300 nM forskolin, both prepared at 4× the desired final concentration in stimulation buffer, were added to a 96-well white ½ area microplate (PerkinElmer). The CHO-hCB1 cells were lifted with a non-enzymatic solution (Cell-stripper, Mediatech Inc., Orlando, FL, USA), and 4000 cells were added to each well. After incubating for 30 min at room temperature, Eu-cAMP tracer and uLIGHT-anti-cAMP working solutions were added per the manufacturer’s instructions. After incubation for 1 h, the TR-FRET signal (ex 337 nm, em 620 and 650 nm) was read on a CLARIO star multimode plate reader (BMG Biotech, Cary, NC, USA). Data were analyzed using Prism software (GraphPad, La Jolla, CA, USA). Nonlinear regression analysis was performed to fit data and obtain maximum response (Emax), EC_50_, correlation coefficient (r^2^), and other parameters. All experiments were performed in duplicate 2–3 times to ensure reproducibility and data are reported as mean ± standard error of mean unless noted otherwise. 

### 4.2. Radioligand Displacement Assay

Further characterization of **21** was performed using radioligand displacement of [^3^H]CP55940 and equilibrium dissociation constant (Ki) value was determined as described previously [27,28]. Data reported are average values from 3 measurements with <30% standard error.

### 4.3. Pharmacokinetic Testing

Female C57BL/6 mice were bred in house and used at ~10 weeks of age for pharmacokinetic (PK) testing. Three animals were tested per time point. Doses were formulated in 2% NMP in canola oil, and all compounds were delivered at 3 mg/kg by intraperitoneal injection (IP). Tissues were taken at 0.5, 1, 2, and 4 h post dose. Animals were subjected to whole body perfusion using saline prior to tissue collection. Brain samples were homogenized with 50:50 ethanol:water (1:5, *v*/*v*). Forty µL of the homogenate, 10 µL of acetonitrile, and 150 µL of 100 ng/mL reserpine in acetonitrile containing 0.1% formic acid were vortexed and centrifuged. Plasma samples were diluted with 10 µL of acetonitrile, 150 µL of 100 ng/mL reserpine in acetonitrile containing 0.1% formic acid, vortexed and centrifuged. Samples were subjected to LC/MS/MS analysis. Standards were prepared in blank samples and used for calibration curves. Chromatography was performed using a Phenomenex Luna C18 column.

### 4.4. Computational Methods

Docking and Induced Fit. Two parallel and independent approaches with different scoring functions, Schrodinger’s XP/Induced Fit [29] and Autodock VINA with an AMBER18 [30] molecular mechanics generalized Born surface area MMGBSA rescoring, were used to provide predictions for ligand configurations allowing for both ligand and receptor binding site flexibility. Both methods include desolvation components in the scoring function. Following initial GLIDE-XP docking, we probed the importance of binding site flexibility within a 5 Å window of any atom in the best docking poses using Schrodinger’s Induced Fit [29]. The docking box employed for VINA was ca 15 × 15 × 15 Å^3^ in spatial extent, while the default box size employed in computing the GLIDE-XP docking grid was based on the ligand size. We employ Autodock VINA’s scoring function scores based on hydrophobic contacts, hydrogen bonds, lack of steric clash. This informatics-based scoring function serves primarily as a means of selecting the most plausible collection of poses from a large number of sampled docked configurations which we rescore using AMBER-MMGBSA. We collected 20 poses per ligand with a high “exhaustiveness” setting of 80. Similarly, the GLIDE-XP workflow and scoring function was used to obtain up to 20 poses per ligand for post-docking minimization but retaining the best Emodel scored 5 poses in the final analysis. The GLIDE-XP scoring function contains terms such as desolvation, lipophilic/hydrophobic contact and cavity costs, in addition to physics based coulombic and van der Waals and ligand strain terms.

For the initial docking/induced-fit and MMGBSA-rescoring phase we chose to use the model completed 5XR8 structure employing simulated annealing with topological/stereochemical constraints in Modeller, followed by SCWRL rotameric sidechain adjustments for loop/terminus modeled residues. We then used AMBER18 employing the AMBER14SB forcefield to prepare the full-length All-H model as described in prior publications [31,32]. The structure prepared with TLEAP was then energy minimized for 8000 steps of conjugate gradient following a 400 steepest-descents minimization to remove initial inferior contacts.

Docked poses above were re-scored using an AMBER 18 MMGBSA and Prime/MMGBSA approaches. This was performed because the top ranked docked poses are often not the crystallographically observed pose as observed by us and others in PDB-Bind assessments [33], whereas frequently the lowest MMGBSA/MMPBSA scored pose has lowest RMSD to the known crystallographic solution and provides the best affinity correlations [34,35,36].

MD simulation. An MD simulation was employed to track pose stability and ligand induced receptor binding site changes employing AMBER18 using ff14SB parameterization for protein residues with GAFF generalized forcefield parameters for internal forcefields of the ligand. ANTECHAMBER and PARMGEN were used to incorporate AM1-BCC charges into the ligand antechamber topology and parameter files. The LIPID14 forcefield was used for parameterization of 150 Å × 150 Å × 143 Å box of DOPC lipids (initially 323 lipids top and bottom). Elastic band pulls of the N− and C− terminal regions and simulated annealing were used to prepare low energy more compact conformations beyond initial extended constructed conformations after the MODELLER/SCWRL preparation steps. The box was used to immerse the CB1/8D0 complex and dynamics was used a method of ‘refining’ the N−/C− and loop conformations following production dynamics. The complex system was then energy minimized by a combination of 800 steepest descents followed by 8000 conjugate gradient steps. The system was then slowly heated to 300K over 200 ps followed by 5 ns of early equilibration under NPT ensemble simulation conditions followed by production dynamics under NVT conditions for 850 ns after establishing equilibration from the standpoint of RMSD variations from the starting structure and energetic and fluctuation criteria.

Native Contact Time Dependent Pharmacophore. Native contact analysis was conducted employing AMBER cpptraj, bash scripting and C++ code to determine the percentage of time of used defined regions in the 8D0/AM841 ligand contacted residues in the binding site. The percentage time of each region of the ligand with amino acids was computed from 850 ns of production dynamics, and a representation of those percentages was presented in a colored heatmap representation.

## Figures and Tables

**Table 1:** Sulfonamide analogs of 1.

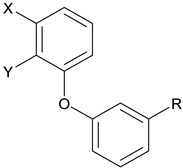					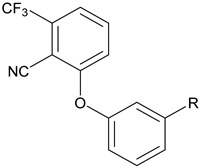		
**#**	**X**	**Y**	**R**	**hCB1 cAMP** **EC**_**50**_ **(nM)** **Efficacy** ^a^	**#**	**R**	**hCB1 cAMP** **EC**_**50**_ **(nM)** **Efficacy** ^**a**^
**1 ^b^**	CF_3_	CN	OSO_2_(CH_2_)_3_CF_3_	46 ± 5 39 ± 11%	**11**	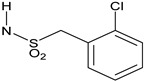	Inactive
**4**	CF_3_	CN	NHSO_2_(CH_2_)_3_CF_3_	190 ± 46 96 ± 5%	**12**	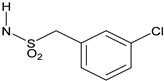	Inactive
**5**	CF_3_	Cl	NHSO_2_(CH_2_)_3_CF_3_	175 ± 42 95 ± 9%	**13**	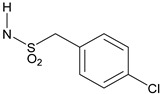	25 ± 4 95 ± 3%
**6**	H	CN	NHSO_2_(CH_2_)_3_CF_3_	106 ± 8 95 ± 8%	**14**	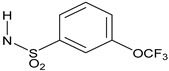	Inactive
**7**	CF_3_	CN	NHSO_2_(CH_2_)_4_Me	248 ± 108 76 ± 20%	**15**	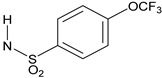	Inactive
**8**	CF_3_	CN	NHSO_2_CH_2_Ph	228 ± 134 102 ± 14%			
**9**	CF_3_	Cl	NHSO_2_CH_2_Ph	255 ± 62 78 ± 17%			
**10**	H	CN	NHSO_2_CH_2_Ph	164 ± 32 82 ± 8%			

^a^ Efficacy was measured as a % of the maximum CP55940 (full agonist) signal in CHO cell membrane preparations overexpressing hCB1 receptor. Data reported as mean ± SEM. ^b^ 1 is BAY 59-3074.

## Data Availability

Not applicable.

## Supplementary Materials

The following supporting information can be downloaded at: https://www.mdpi.com/article/10.3390/molecules27175672/s1, Table S1. Calculated Properties of Compounds: MW, cLogP, TPSA, HBD, and HBA, Figures S1–S5. HPLC and NMR data of key compounds.

## Sample Availability

Samples of the compounds are available from the authors.

## Abbreviations

cAMP, cyclic adenosine monophosphate; CB1, cannabinoid receptor 1; CB2, cannabinoid receptor 2; CHO, Chinese hamster ovary cells; CNS, central nervous system; DMA, dimethylacetamide; DMF, dimethylformamide; EtOH, ethanol; HPLC, high pressure liquid chromatography; GI, gastrointestinal; IP, intraperitoneal injection; PK, pharmacokinetic; MS, mass spectroscopy; MW, molecular weight; NASH, nonalcoholic steatohepatitis; NMR, nuclear magnetic resonance; rt, room temperature; SAR, structure–activity relationship; TFA, trifluoroacetic acid; THC, (−)-trans-Δ^9^-Tetrahydrocannabinol; THF, tetrahydrofuran; TLC, thin layer chromatography; TPSA, topological polar surface area; UV, ultraviolet.
