# Supplementary material for: Structure–Activity Relationship Development Efforts towards Peripherally Selective Analogs of the Cannabinoid Receptor Partial Agonist BAY 59-3074
Source: Molecules. 2022 Sep 2;27(17):5672. doi: 10.3390/molecules27175672 (PMC9457575; doi:10.3390/molecules27175672)

Resubmission Molecules (MS# molecules - 1866652)

Structure Activity Relationship Development Efforts Towards Peripherally Selective Analogs of the Cannabinoid Receptor Partial Agonist BAY 59-3074

George Amato, Vineetha Vasukuttan, Danni Harris, Lucas Laudermilk, Jennifer Lucitti, Scott Runyon and Rangan Maitra*

* Corresponding author

Center for Drug Discovery | RTI International

P.O. Box 12194 | Research Triangle Park, NC 27709-2194, USA

Phone: (+1) 919-541-6795 | Web: https://goo.gl/4uidz7

Table S1. Calculated Properties of Compounds					
**#**	**MW[a]**	**cLogP[b]**	**TPSA[b]**	**HBD**	**HBA**
**1**	453	5.1	76	0	5
**4**	452	4.2	79	1	5
**5**	462	5.0	55	1	5
**6**	384	3.4	79	1	5
**7**	412	4.5	79	1	5
**8**	432	4.5	79	1	5
**9**	442	5.2	55	1	5
**10**	364	3.6	79	1	5
**11**	467	5.1	79	1	5
**12**	467	5.1	79	1	5
**13**	467	5.1	79	1	5
**14**	502	6.1	88	1	6
**15**	502	6.1	88	1	6
**16**	376	5.5	62	1	4
**17**	378	5.4	71	1	5
**18**	377	4.7	74	2	6
**19**	391	4.9	65	1	6

**20**	396	5.3	62	1	4
**21**	439	4.2	91	2	6
**22**	412	4.5	79	1	5
**23**	432	4.5	79	1	5
**24**	396	5.3	62	1	4

a. Calculated with ChemDraw suite.

b. Calculated with ChemAxon suite.

Figure S1. Compound 21 HPLC

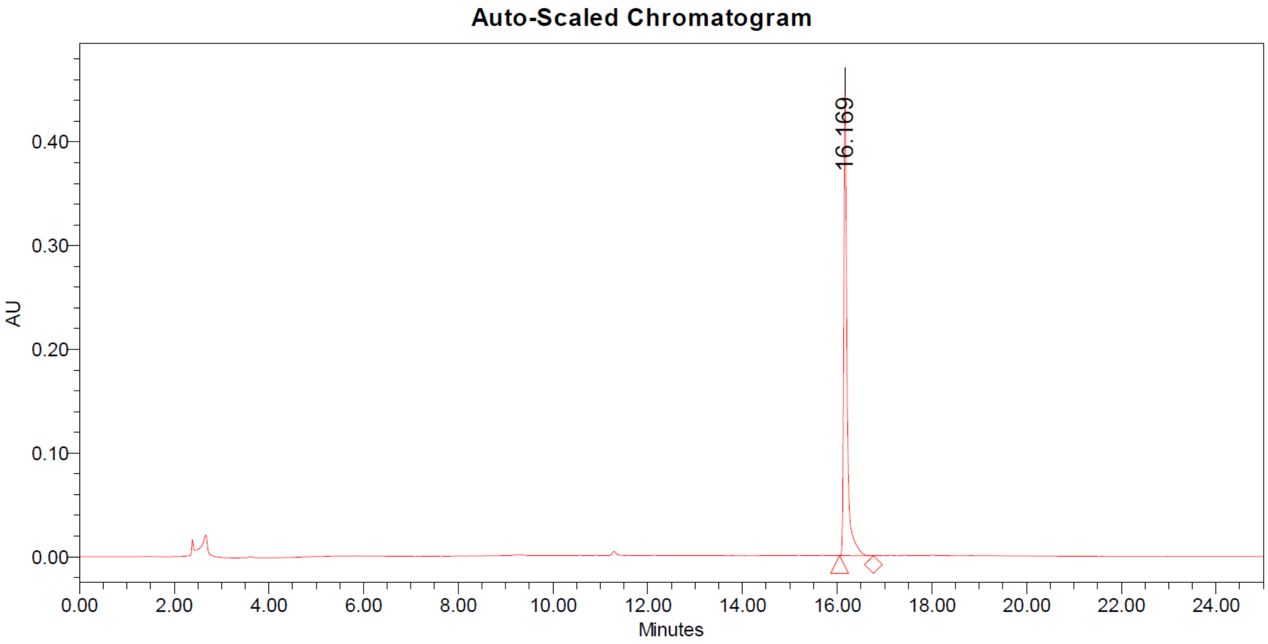

Peak Results

	RT	Area	% Area	Height
1	16.169	2196004	100.00	453142

Figure S2. Compound 21 ^{1}H NMR spectra

^{1}H NMR (300 MHz, CHLOROFORM-*d*) δ ppm 7.53 - 7.70 (m, 1 H), 7.47 (d, *J*=7.54 Hz, 1 H), 7.36 (t, *J*=8.19 Hz, 1 H), 7.08 (d, *J*=8.48 Hz, 1 H), 7.00 (br. s., 3 H), 6.81 (d, *J*=7.54 Hz, 1 H), 4.66 (d, *J*=6.78 Hz, 1 H), 3.13 - 3.38 (m, 1 H), 1.77 - 1.96 (m, 2 H), 1.65 (br. s., 3 H), 1.47 - 1.59 (m, 1 H), 1.06 - 1.37 (m, 6 H)

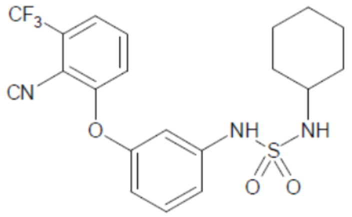

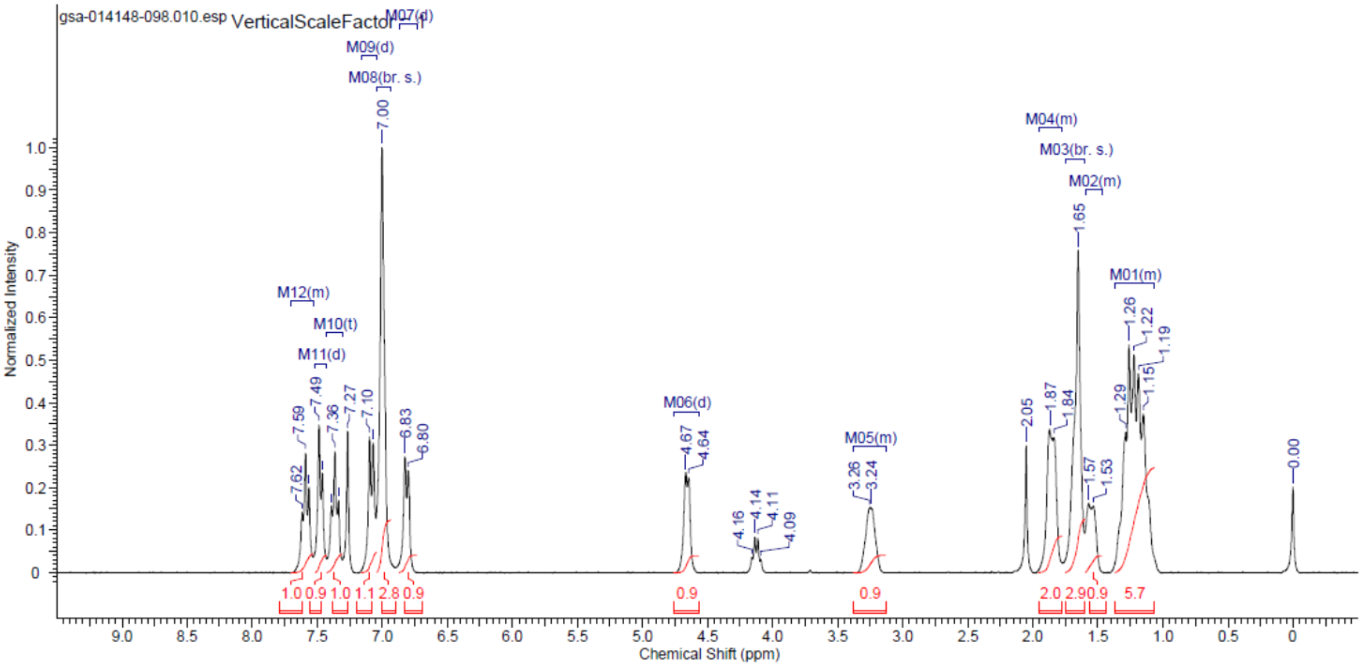

Figure S3. Compound 21 ^{13}C NMR spectra

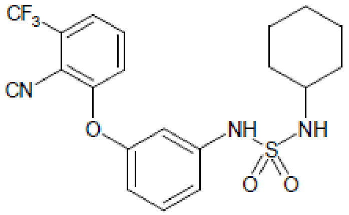

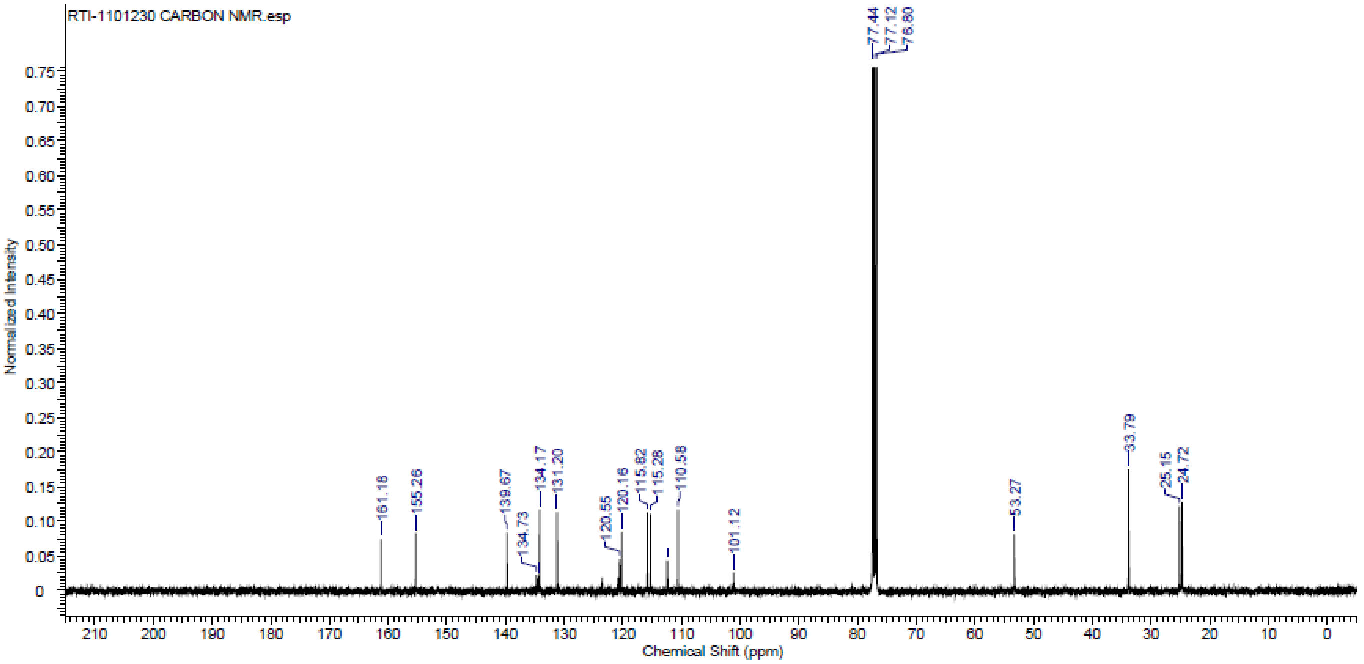

Figure S4. Compound 24 HPLC

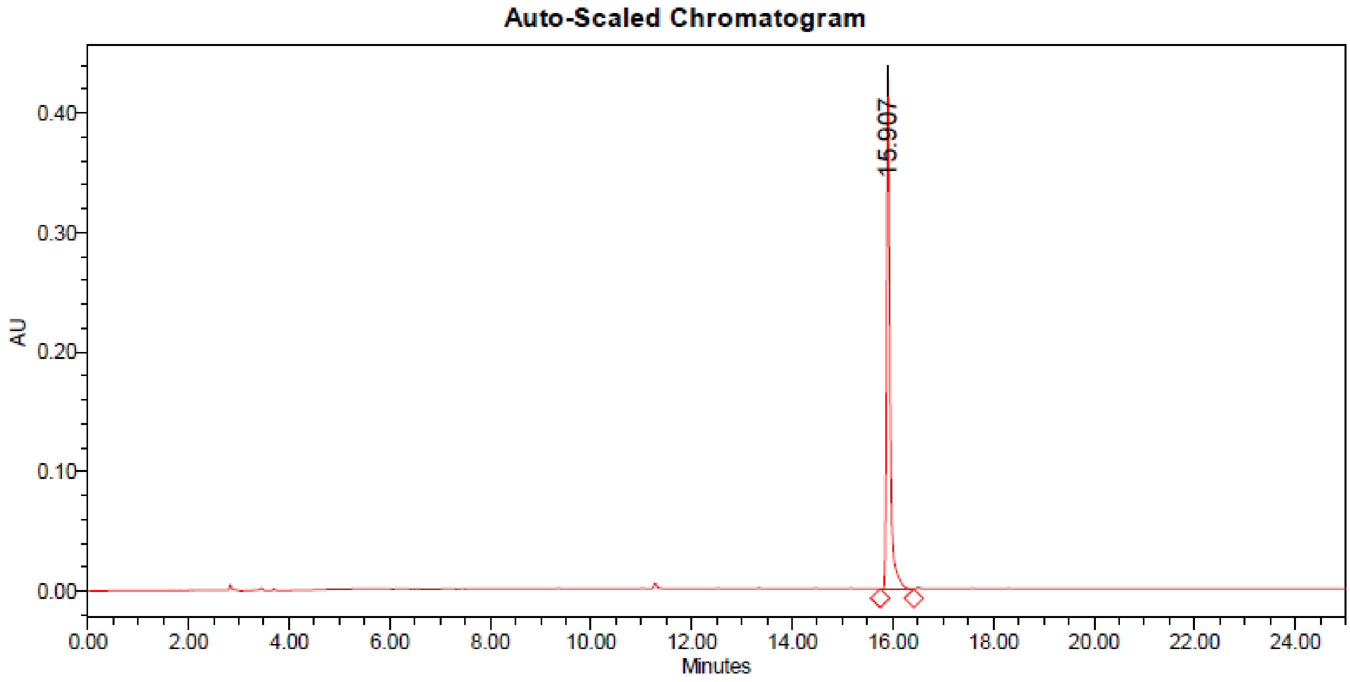

Peak Results

	RT	Area	% Area	Height
1	15.907	2037104	100.00	425766

Figure S5. Compound 24 ^{1}H NMR spectra

^{1}H NMR (300 MHz, CHLOROFORM-*d*) δ ppm 7.46 - 7.60 (m, 3 H), 7.37 (s, 3 H), 7.41 (s, 3 H), 7.19 (br. s., 1 H), 6.93 - 7.11 (m, 3 H), 3.77 (br. s., 2 H)

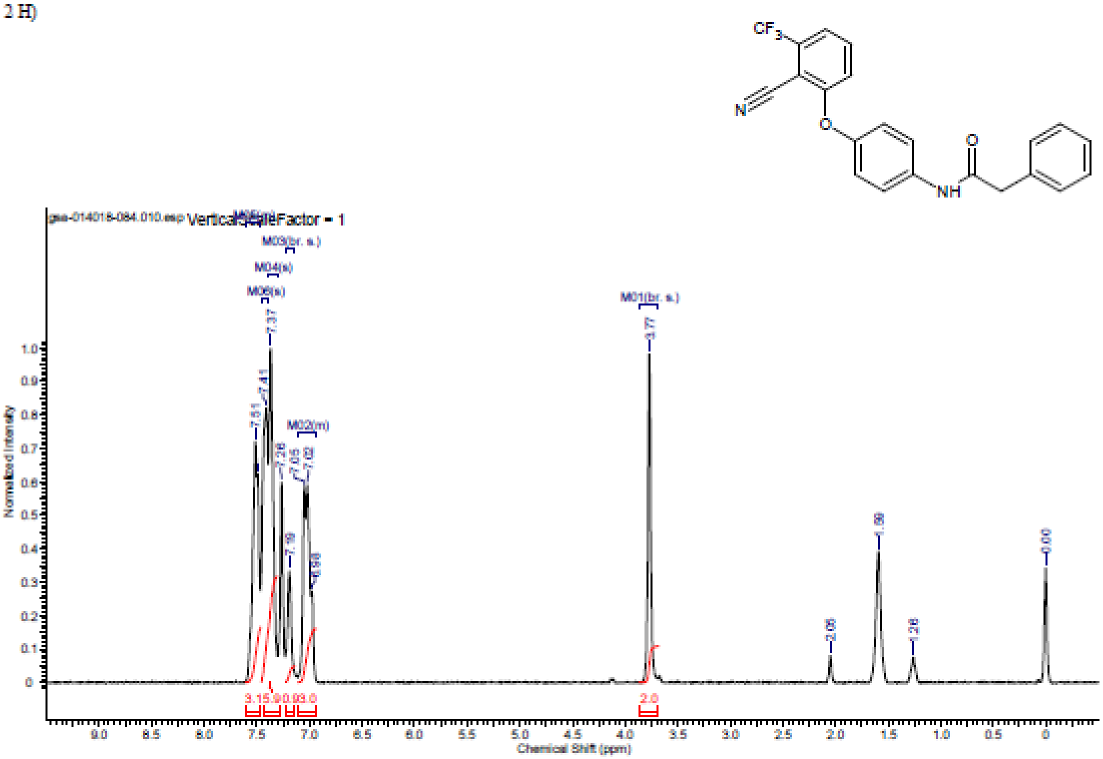

Supplement: Supplementary file 1 [file molecules-27-05672-s001.zip › molecules-1866652-supplementary.pdf]
